# A dataset for translating local Bangla (Sylheti) dialects into standard Bangla

**DOI:** 10.1016/j.dib.2026.112576

**Published:** 2026-02-13

**Authors:** Tabia Tanzin Prama, Mangsura Kabir Oni

**Affiliations:** Jahangirnagar University, Dhaka 1342, Bangladesh

**Keywords:** Sylheti dialect, Standard Bangla, Parallel corpus, Machine translation, Natural language processing

## Abstract

Sylheti is a language spoken by about 11 million people worldwide. It's mostly spoken in northeastern Bangladesh and southern Assam, India, and by people living in other countries who originally came from these regions. Translating Sylheti dialects into Standard Bangla is essential to ensure effective communication across the country and internationally. This article introduces a collection of paired sentences, one in the Sylheti dialect and the other in Standard Bangla. It was created to enhance Neural Machine Translation (NMT) between the two languages. Sylheti is a language with a rich cultural heritage, known for its unique vocabulary, music, and folklore. However, it has been largely absent from formal written materials and digital resources, leaving a gap in its linguistic representation. To bridge this gap, 5002 sentence pairs were carefully collected from various sources, such as Bangladeshi newspapers, social media platforms, voluntary comments and contributions from native Sylheti speakers. The dataset, collected between December 2023 and March 2025, contains diverse linguistic elements. It includes 21,132 unique words (9729 Sylheti words and 11,403 Standard Bangla words), 10,340 clauses (5069 Sylheti and 5271 Standard Bangla), and 10,004 sentences. This collection is not only valuable for machine translation but also plays a crucial role in other areas of natural language processing. It supports tasks like text classification, identifying key names and entities, and analyzing sentiment. Furthermore, it enables the development of advanced technologies for Sylheti, such as text-to-speech systems, sentiment analysis tools, and language models. This resource is a significant step towards better understanding and utilizing the Sylheti language in the digital world.

Specifications Table

**Table:** 

Subject	Computer Science, Artificial Intelligence
Specific subject area	Translation of Sylheti Dialects into Standard Bengali Dialect, Natural Language Processing, Human Language Technology, Linguistic corpus
Data Format	Text/String, Raw
Type of data	Table (.csv format)
Data collection	The dataset [1] was created by combining data from various sources, including Bangladeshi national newspapers, social media posts, and comments from the Sylheti-speaking community, with their consent, confirming that they understood the objectives of the study, the significance of their participation, and their right to discontinue participation at any moment. Even the dialogues and conversations from Sylheti regional entertaining drama were stored. As part of the data collection process, Sylheti dialect sentences were translated into equivalent standard Bangla sentences. Additionally, Sylheti rhymes were translated into standard Bangla to further enrich the dataset.
Data source location	**Institution:** Various sources, including Bangladeshi national newspapers (e.g., *Prothom Alo, The Daily Star, Ittefaq*), social media platforms (e.g., Facebook, Twitter, Instagram, YouTube), voluntary comments, the Sylheti-speaking community, and Sylheti literature and folklore archives. **City/Region:** Jahangirnagar University, Savar, Dhaka-1342 **Country:** Bangladesh
Data accessibility	Repository name: Mendeley DataData identification number: 10.17632/5rmskrvh6g.1Direct URL to data: https://data.mendeley.com/datasets/5rmskrvh6g/1Instructions for accessing these data: 1. Go to the provided data availability section. 2. Locate the .csv file. The file contains two columns: one for Local Bangla Dialect (Sylheti) and another for Standard Bangla Text. 3. Directly download and access the dataset from there.
Related research article	None

## 1 Value of the Data

• This dataset captures the most frequently used Sylheti words, clauses, and sentences which supports the development and training of machine learning models for tasks such as machine translation, text classification, sentiment analysis, and named entity recognition.
• Currently, translating Sylheti to Bengali is done by people, which takes a long time and can have mistaken. This dataset helps computers learn to translate accurately and consistently. It teaches computers about the special features of the Sylheti language, helping them understand and process it better.
• This dataset is useful for building applications that empower Sylheti-speaking communities with tools for communication, cultural preservation, and digital accessibility.
• It improves how people who speak Sylheti can communicate with others, especially in fields like farming, treatment and education.
• This dataset helps to keep the Sylheti language alive and accessible for future generations.
• Consists of 1200 parallel sentence pairs in Sylheti and Standard Bangla, addressing the critical lack of linguistic resources for the Sylheti dialect. It supports NLP by providing a strong base for creating efficient and scalable models for Sylheti and Standard Bangla.

## 2 Background

Sylheti, known for its rich vocabulary, music, and folklore, is mainly an oral dialect with limited written forms. Unlike Standard Bangla, which dominates documentation and digital platforms, Sylheti lacks resources, leaving its speakers behind in modern technology. This gap has slowed the development of NLP tools for Sylheti. Though an Eastern Indo-Aryan language in the Bengali-Assamese continuum, Sylheti is not officially recognized in Bangladesh or India. Often seen as a dialect of Bengali, it is dismissed by some as slang. In Bangladesh, Standard Bangla is the sole official language and holds significant political importance [2]. This highlights the need for translating Sylheti into Standard Bangla to ensure better communication and representation.

Gaustad [3] created a dataset for Siswati, a Bantu language spoken in Eswatini and South Africa. Siswati is one of South Africa's eleven official languages and is spoken alongside English in Eswatini. The dataset includes parallel text data in English and Siswati, as well as monolingual Siswati data. It was developed to help train machine translation systems, particularly for the Autshumato project. In another study, Sultana [4] proposed a dialect into Standard Bangla using Neural Machine Translation (NMT) system and their dataset includes a parallel corpus with 1540 words, 130 clauses, and 980 sentences for each dialect, along with their Standard Bangla and English translations. Nerbonne [5] collected data from *Reeks Nederlands(ch)e Dialectatlassen* to compare and classify dialects. The dataset includes 1956 transcriptions of 141 sentences from 104 dialects across the Dutch-speaking area, with 100 words chosen to cover all vowels and consonants in each dialect, these are some research studies about dialects conversion system. A recent study, Prama [6] has worked on converting the Sylheti dialect to standard Bangla using a neural machine translation system. This study employs deep neural networks to achieve the translation. However, the dataset used in this research is not yet publicly available. Our corpus provides Sylheti speakers with access to advanced language technologies, helping to include their dialect in the digital world. By addressing the lack of written and digital resources, it plays a key role in preserving linguistic diversity and supporting the cultural identity of underserved communities.

## 3 Data Description

**Fig. 1:**
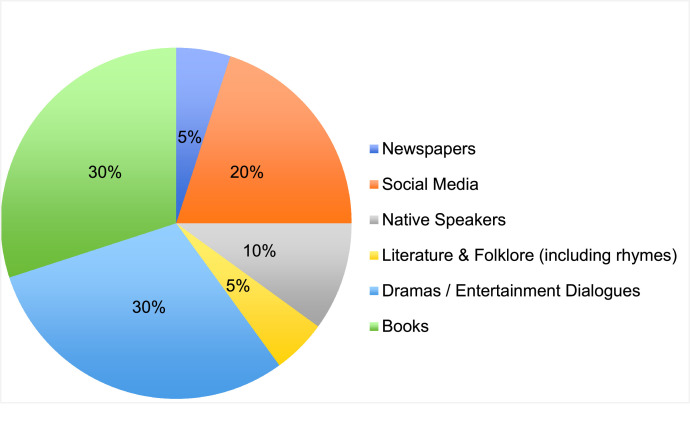
Distribution of data sources in the Sylheti–Standard Bangla parallel corpus.

To create a diverse and comprehensive parallel corpus of Sylheti and Standard Bangla sentences, we undertook an extensive data collection process from December 2023 to January 2025. The dataset was developed to address the critical lack of linguistic resources for the Sylheti dialect, a historically significant yet underrepresented language. We gathered a total of 5002 bilingual sentence pairs, representing authentic Sylheti dialect sentences alongside their Standard Bangla translations. The primary data sources included widely recognized Bangladeshi national newspapers, social media platforms and contributions from native Sylheti speakers. The data collection process included curating Sylheti sentences from books,^1^ news articles, online posts, comments, and folk rhymes. For example, sentences were collected from prominent newspapers such as *Prothom Alo, The Daily Star*, and *Ittefaq*. Additionally, data was sourced from platforms like Facebook, Twitter, Instagram, and YouTube, ensuring diverse linguistic and cultural representation. A significant portion of the dataset was further enriched with contributions from Sylheti-speaking community members residing in Dhaka, Sylhet, and other regions, as well as from Sylheti literature and folklore archives. The transparent distribution of data sources is illustrated in Fig. 1.

**Fig. 2:**
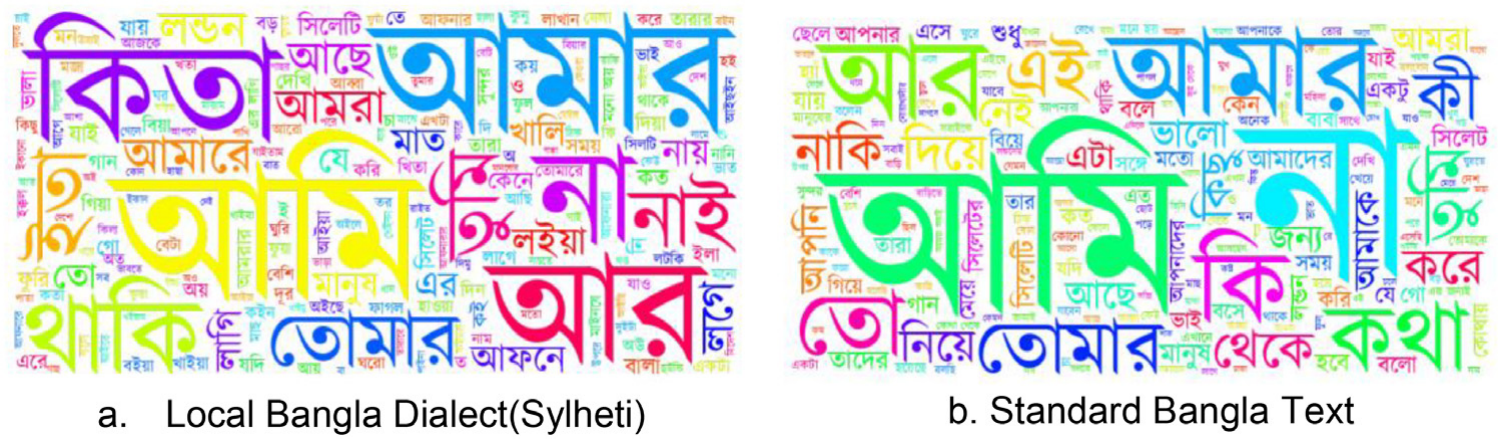
Word cloud of local Bangla dialect and standard Bangla text from the dataset.

Table 1 shows some examples of our dataset and Table 2 provides a summary of the dataset's structure, ensuring its readiness for computational tasks. Fig. 2 presents the word cloud of the dataset, where Fig. 2(a) represents the Sylheti dialect dataset, and Fig. 2(b) represents the Standard Bangla dataset. Although both word clouds convey similar meanings, the differences in dialects lead to variations in pronunciation and word usage.

**Table 1:** Examples of the dataset.

	Category	
	Sylheti Language	Standard Bangla Language
		
**Word**	আখতা	হঠাৎ
**Clause**	আইলর ঘাস	আঁইলের ঘাস
**Sentence**	শীতর হাওয়া আমারে ছইয়া যায়	শীতের হাওয়া আমাকে ছুঁয়ে যায়

**Table 2:** Descriptive statistics of the dataset.

	**Category**		**Total**
	Sylheti	Standard Bangla	
**Word**	9729	11,403	21,132
**Clause**	5069	5271	10,340
**Sentence**	5002	5002	10,004

## 4 Experimental Design, Materials and Methods

**Fig. 3:**
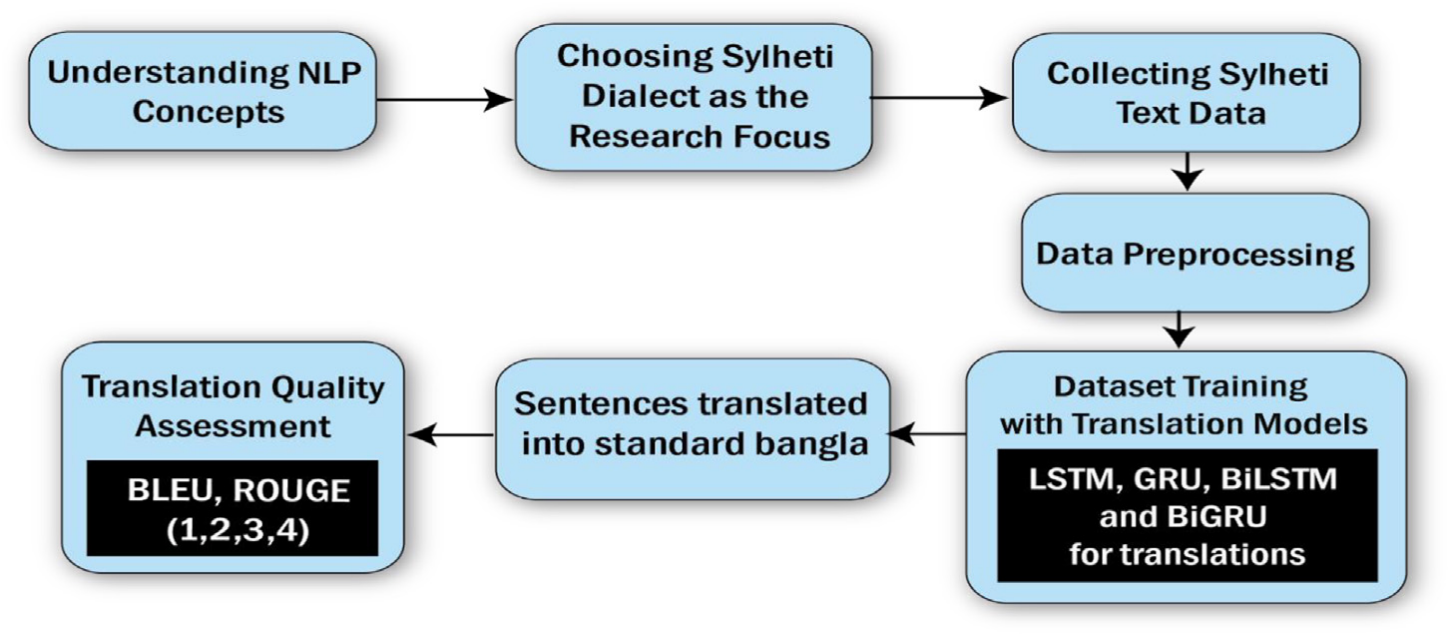
The proposed workflow diagram of the dataset.

The Fig. 3 presents a structured workflow for processing the Sylheti dialect and translating it into Standard Bangla, focusing on the use of Natural Language Processing (NLP) techniques. The process begins with gaining a solid understanding of key NLP concepts, including tokenization, text classification, and machine translation, which are essential for handling low-resource languages like Sylheti. Next, Sylheti is selected as the primary focus for research, recognizing its cultural importance and the significant lack of linguistic resources in digital platforms. Data collection forms a crucial step, where Sylheti text is gathered from diverse sources such as oral histories, social media posts, and written documents. Native speakers play an essential role in this process, ensuring the authenticity and accuracy of the collected data. The collected text then undergoes detailed preprocessing, which includes removing unnecessary punctuation, special characters, and irrelevant symbols. Further steps such as tokenization (breaking sentences into words or tokens), normalization of spelling, and alignment of Sylheti-Bangla sentence pairs are carried out to prepare the data for machine learning tasks. This preprocessing ensures that the dataset is clean, well-structured, and ready for use in translation models. During data collection and preprocessing, several challenges were encountered:

• The limited availability of written Sylheti materials in formal sources such as newspapers and online media required reliance on social platforms and community contributions.
• Many sentences had to be manually translated and carefully validated by native speakers to ensure both linguistic accuracy and cultural appropriateness.
• The wide variation in Sylheti spelling and usage across different speakers introduced inconsistencies that required normalization during preprocessing.
• Idiomatic expressions and culturally embedded phrases often lacked direct equivalents in Standard Bangla, which required context-aware interpretation and careful handling.

Once the data is prepared, Sylheti sentences are translated into Standard Bangla to create a parallel bilingual corpus. This dataset serves as the foundation for training advanced translation models, such as Long Short-Term Memory (LSTM), Gated Recurrent Units (GRU), Bidirectional LSTM (BiLSTM), and Bidirectional GRU (BiGRU). These models are carefully trained to understand the unique features of Sylheti and produce accurate translations into Standard Bangla.

The quality of translations is evaluated using standard metrics such as BLEU (BLEU-1, BLEU-2, BLEU-3, BLEU-4) and ROUGE (ROUGE-1, ROUGE-2, ROUGE-3, ROUGE-4), which assess how closely the machine-generated translations align with human translations. These metrics help ensure the reliability and accuracy of the models in producing meaningful translations. Overall, this approach combines linguistic expertise with machine learning techniques to develop effective language tools, preserving the unique characteristics of Sylheti while bridging the gap between this lesser-known dialect and Standard Bangla.

## Limitations

Our Sylheti-Standard Bangla corpus is useful but has some limitations. It contains 5002 sentence pairs, which may not be enough for training highly accurate models, especially for complex tasks like machine translation. The dataset's size could affect the model's ability to handle diverse, unseen data. While the data was collected from newspapers, social media, and community contributions, certain dialects, regions, or genres may be overrepresented, potentially introducing biases that could affect model performance. Handling subtle dialectal variations and accurately translating idiomatic or culturally embedded expressions remains challenging, often requiring careful manual validation by native speakers. Furthermore, similar to findings in low-resource language corpora such as KurdSM [7], the limited availability of high-quality, diverse sources can constrain the representativeness of the dataset. Future work could benefit from additional annotations, broader coverage of dialects, and more varied linguistic contexts to enhance the corpus for advanced NLP tasks.

## Ethics Statement

Our research involved close collaboration with native Sylheti speakers, who contributed valuable insights and data for this project. Prior to participation, informed consent was obtained from all contributors. They were provided with a clear explanation of the study’s objectives, the potential impact of their contributions, and their right to withdraw at any time without any consequences. All social media data used in the dataset were carefully anonymized to protect user privacy. This process involved systematically removing personal identifiers such as usernames, profile names, phone numbers, email addresses, and location information. Any content that could reveal the identity of individuals, including images, videos, or links, was excluded. Only textual content directly relevant to Sylheti sentences was retained. Posts or comments containing sensitive, offensive, or potentially harmful material were also removed. To ensure the accuracy and cultural appropriateness of translations, native Sylheti speakers reviewed and validated all sentence pairs. Throughout the entire process, we maintained strict adherence to ethical research standards, ensuring that the dataset contains only linguistic material, respects participants’ privacy, and preserves the cultural integrity of the Sylheti dialect.

## Declaration of generative AI and AI-assisted technologies in the writing process

During the preparation of this work the author(s) used ChatGPT (OpenAI) to improve language and readability. After using this tool, the author(s) reviewed and edited the content as needed and take(s) full responsibility for the content of the publication.

## CRediT authorship contribution statement

**Tabia Tanzin Prama:** Visualization, Conceptualization, Methodology, Investigation, Supervision, Validation, Writing – review & editing. **Mangsura Kabir Oni:** Visualization, Conceptualization, Methodology, Software, Data curation, Writing – original draft.

## Data Availability

(Mendeley Data).A Dataset for Translating Local Bangla (Sylheti) Dialects into Standard Bangla (Original data)

## Footnotes

1 আহমেদ হাসান (সম্পাদক). সিলেটের উপভাষার শব্দকোষ. ১ম প্রকাশনা, প্রতিভা প্রকাশ, ২০১৭, ISBN: 9789849260530.
